# Benefit-risk profile of cytoreductive drugs along with antiplatelet and antithrombotic therapy after transient ischemic attack or ischemic stroke in myeloproliferative neoplasms

**DOI:** 10.1038/s41408-018-0048-9

**Published:** 2018-02-28

**Authors:** Valerio De Stefano, Alessandra Carobbio, Vincenzo Di Lazzaro, Paola Guglielmelli, Alessandra Iurlo, Maria Chiara Finazzi, Elisa Rumi, Francisco Cervantes, Elena Maria Elli, Maria Luigia Randi, Martin Griesshammer, Francesca Palandri, Massimiliano Bonifacio, Juan-Carlos Hernandez-Boluda, Rossella Cacciola, Palova Miroslava, Giuseppe Carli, Eloise Beggiato, Martin H. Ellis, Caterina Musolino, Gianluca Gaidano, Davide Rapezzi, Alessia Tieghi, Francesca Lunghi, Giuseppe Gaetano Loscocco, Daniele Cattaneo, Agostino Cortelezzi, Silvia Betti, Elena Rossi, Guido Finazzi, Bruno Censori, Mario Cazzola, Marta Bellini, Eduardo Arellano-Rodrigo, Irene Bertozzi, Parvis Sadjadian, Nicola Vianelli, Luigi Scaffidi, Montse Gomez, Emma Cacciola, Alessandro M. Vannucchi, Tiziano Barbui

**Affiliations:** 1 Institute of Hematology, Catholic University, Fondazione Policlinico Universitario A. Gemelli, Roma, Italy; 2 FROM Research Foundation, Papa Giovanni XXIII hospital, Bergamo, Italy; 3 Unit of Neurology, Neurophysiology, Neurobiology, Department of Medicine, Università Campus Biomedico di Roma, Rome, Italy; 4 CRIMM-Center of Research and Innovation of Myeloproliferative Neoplasms, Azienda Ospedaliera Universitaria Careggi, and Departmentt Experimental and Clinical Medicine, University of Florence, Firenze, Italy; 5 Hematology Division, IRCCS Ca’ Granda - Maggiore Policlinico Hospital Foundation, and University of Milan, Milan, Italy; 6 Hematology Division, Papa Giovanni XXIII hospital, Bergamo, Italy; 7 Department of Molecular Medicine, University of Pavia, Pavia, Italy; 8 Department of Hematology Oncology, Fondazione IRCCS Policlinico San Matteo, Pavia, Italy; 9 Hospital Clínic, IDIBAPS, University of Barcelona, Barcelona, Spain; 10 Hematology Division, Ospedale San Gerardo, ASST Monza, Monza, Italy; 11 Department of Medicine - DIMED, University of Padua, Padova, Italy; 12 University Clinic for Hematology and Oncology Minden, University of Bochum, Bochum, Germany; 13 Institute of Hematology “L. and A. Seràgnoli”, S. Orsola-Malpighi Hospital, Bologna, Italy; 14 Department of Medicine, Section of Hematology, University of Verona, Verona, Italy; 15 Hematology Department, Hospital Clínico Universitario, Valencia, Spain; 16 Department of Clinical and Experimental Medicine, University of Catania, Catania, Italy; 17 Department of Hemato-Oncology, Faculty of Medicine and Dentistry, Palacky University Olomouc, Olomouc, Czech Republic; 18 Hematology Department, Ospedale San Bortolo, Vicenza, Italy; 19 Unit of Hematology, Department of Oncology, University of Torino, Torino, Italy; 20 Hematology Institute and Blood Bank, Meir Medical Center, Kfar Saba, and Sackler School of Medicine Tel Aviv University, Tel Aviv, Israel; 21 Division of Hematology, Dipartimento di Patologia Umana dell’Adulto e dell’Età Evolutiva, Policlinico G Martino, University of Messina, Messina, Italy; 22 Division of Hematology, Department of Translational Medicine, University of Eastern Piedmont, Novara, Italy; 23 S.C. Ematologia, Azienda Ospedaliera S. Croce e Carle, Cuneo, Italy; 24 Divisione di Ematologia, Arcispedale Santa Maria Nuova-IRCCS, Reggio Emilia, Italy; 25 Hematology and Bone Marrow Transplantation Unit, IRCCS San Raffaele Scientific Institute, Milano, Italy; 26 Neurology Division, Papa Giovanni XXIII hospital, Bergamo, Italy; 27 Department of Medical, Surgical and Advanced Technologies Sciences “G.F. Ingrassia”, University of Catania, Catania, Italy

## Abstract

We analyzed 597 patients with myeloproliferative neoplasms (MPN) who presented transient ischemic attacks (TIA, *n* = 270) or ischemic stroke (IS, *n* = 327). Treatment included aspirin, oral anticoagulants, and cytoreductive drugs. The composite incidence of recurrent TIA and IS, acute myocardial infarction (AMI), and cardiovascular (CV) death was 4.21 and 19.2%, respectively at one and five years after the index event, an estimate unexpectedly lower than reported in the general population. Patients tended to replicate the first clinical manifestation (hazard ratio, HR: 2.41 and 4.41 for recurrent TIA and IS, respectively); additional factors for recurrent TIA were previous TIA (HR: 3.40) and microvascular disturbances (HR: 2.30); for recurrent IS arterial hypertension (HR: 4.24) and IS occurrence after MPN diagnosis (HR: 4.47). CV mortality was predicted by age over 60 years (HR: 3.98), an index IS (HR: 3.61), and the occurrence of index events after MPN diagnosis (HR: 2.62). Cytoreductive therapy was a strong protective factor (HR: 0.24). The rate of major bleeding was similar to the general population (0.90 per 100 patient-years). In conclusion, the long-term clinical outcome after TIA and IS in MPN appears even more favorable than in the general population, suggesting an advantageous benefit-risk profile of antithrombotic and cytoreductive treatment.

## Introduction

Polycythemia vera (PV), essential thrombocythemia (ET), and primary myelofibrosis (PMF) are chronic myeloproliferative neoplasms (MPNs) characterized by clonal expansion of an abnormal haematopoietic stem/progenitor cell^1^ that harbours somatic gene mutations: *JAK2* mutations define PV, while mutations of calreticulin (*CALR*) and thrombopoietin receptor (*MPL*) are specific to the majority of *JAK2* un-mutated ET and PMF^2^.

The natural history of these disorders is marked by venous and arterial thrombosis, hemorrhagic complications, and a propensity to transform into myelofibrosis (MF) and acute myeloid leukaemia (AML)^2^. Arterial thrombosis accounts for approximately two thirds of all major thrombotic events and represents a major cause of mortality. In particular, ischemic stroke (IS) and transient ischemic attack (TIA) are most prevalent in comparison with acute myocardial infarction (AMI) and peripheral artery occlusions^3,4^.

The incidence of ischemic cerebrovascular events (ICVE) in MPN has been evaluated in a few studies. In a cohort of 1638 PV patients enrolled in the European Collaborative Low-dose Aspirin Polycythemia Vera (ECLAP) study^5^ and in a recent large observational study, including 1545 patients with *JAK2-*mutated PV^6^, the incidence was 1.2 and 0.9 per 100 patient-years, respectively. Similar findings were reported in ET patients. In the PT-1^7^ and in the ANA-HYDRET^8^ trials, the incidence of ICVE was 1.2 and 1.7 per 100 patient-years, respectively. These rates are approximately 10-fold higher than in the general population, where the incidence is estimated at 0.17 per 100 patient-years^9^.

Patients with MPN presenting with any type of venous and arterial thrombosis or with a previous history of cardiovascular (CV) events are labelled at high-risk for recurrences, and cytoreduction in addition to antithrombotic drugs is recommended to reduce the rate of recurrence^10–12^, although both the modalities of treatment and the effectiveness on prevention of recurrences have been scarcely explored. In a single retrospective study, recurrence after TIA/stroke in MPN was estimated at 1.18 per 100 patient-years^13^. This figure is even lower than in the general population in which the long-term incidence of recurrent IS varies from 2.0 to 3.0 per 100 patient-years^14–17^. Moreover, there is no controlled study assessing whether the incidence of major bleeding in MPN patients, eventually attributable to the use of antithrombotic drugs, is higher than in the general population owing the intrinsic bleeding characteristics of MPNs^3^.

Therefore, considering the scarcity of data on the benefit/risk ratio of prophylaxis of recurrence with antithrombotic and cytoreductive drugs after those life-threatening complications, we launched an international investigator-driven retrospective cohort study called the Preventing Ischaemic Stroke in Myeloproliferative Neoplasms (PRISM) study with three purposes: to describe the clinical profile at presentation of TIA/IS in contemporary PV, ET, and PMF patients; to evaluate the short and long-term outcomes beyond the acute phase, also in comparison with the general, non-MPN population; and to refine the risk assessment for recurrences and CV mortality.

## Patients and methods

### Study patients

A retrospective cohort study was conducted across 22 sites of 5 countries in the European Union and Israel within the European Leukaemia Network (ELN) in patients with a diagnosis of MPN according to the WHO 2008 criteria, after approval of the ethic committees (ECs) (primary approval by the central EC of the coordinating centre was obtained on 12 May 2016).

The participants were asked to identify, among all patients with MPN, those who had suffered from TIA and IS in the period from January 1, 2005 to December 31, 2015. The index event had to be concurrent with, or in the 2 years before, MPN diagnosis, or occurring during the course of a previously diagnosed MPN disease.

Stroke was defined as an episode of acute neurological dysfunction caused by focal cerebral ischaemia, based on objective imaging techniques (computed tomography scan or magnetic resonance imaging scan) and clinical evidence of cerebral focal ischemic injury based on symptoms of any duration. TIA was defined as a transient episode of neurological dysfunction caused by focal brain ischemia, presenting with a unilateral motor or sensory deficit, speech deficit, haemianopsia or monocular blindness, lasting less than 24 h and without evidence of acute infarction on neuroimaging. Patients with hemorrhagic stroke (both intra-parenchymal and subarachnoid haemorrhage), cerebral venous thrombosis, and iatrogenic stroke (i.e., strokes related to neuro-endovascular procedures or coronary angiography) were excluded. Two neurologists (VDL and BC) independently reviewed the diagnostic criteria employed to record TIA and IS patients.

Treatment in the acute phase was delivered by stroke specialists after consulting with haematologists who, after the acute event, took care of these patients during the follow-up.

Each centre reported patients’ information by data input into an electronic database developed to record all study data after de-identification of the patients with an alphanumeric code to protect personal privacy. For each patient, the following information was recorded: demographic data, WHO diagnosis, type of ICVE, method of objective diagnosis, presence of microvascular disturbances or constitutional symptoms, mutation status, the results of the laboratory investigation for inherited or acquired thrombophilia (that is, deficiency of antitrombin, protein C, protein S, factor V Leiden, prothrombin G20210A, increased levels of factor VIII, hyperhomocysteinemia, lupus anticoagulant, anticardiolipin, and anti-beta2-glycoprotein I antibodies), full blood count at diagnosis and at thrombosis, presence of CV risk factors (that is, history of previous thrombosis before the index event, smoking habit, hypertension and dyslipidaemia, diabetes, congestive heart failure) and risk factors for cardiogenic embolism or vascular embolism (that is, atrial fibrillation, patent foramen ovale, valvular heart disease, significant carotid artery stenosis). Microvascular disturbances included erythromelalgia, scintillating scotoma, pulsatile headache, dizziness, and tinnitus. Diagnosis of microvascular disturbances, as well as of TIA, was accepted only in the presence of unequivocal medical documentation.

Data regarding cytoreductive or antithrombotic treatment after index IS or TIA, duration of the treatment and reasons for discontinuation of the treatments were recorded. Finally, follow-up data concerning novel thrombotic events, bleeding complications, MPN haematologic evolution, and death were also recorded.

### Outcomes after the index event

Recurrent IS was defined as a focal neurologic deficit of any duration with evidence at neuroimaging of brain ischemia located in an area not corresponding to the lesion(s) of the index stroke. Recurrent TIA was defined as a novel transient neurologic dysfunction (duration of less than 24 h) without evidence of infarction on brain imaging. Two neurologists (VDL and BC) independently reviewed and validated diagnoses of novel TIA and IS after the index event.

Other arterial or venous thrombotic events that occurred after the index event were recorded only if objectively documented. Arterial recurrences besides ICVE included AMI, unstable angina pectoris and peripheral arterial thrombosis. Objectively established deep venous thrombosis (DVT) of the legs and pulmonary embolism (PE), DVT of the arm, occlusion of cerebral or abdominal veins, thrombosis of the great saphenous vein of the leg objectively diagnosed with ultrasonography, and retinal vein thrombosis were also computed after the index TIA/IS.

Major bleeding was defined according to the criteria of the International Society on Thrombosis and Haemostasis^18^ and included fatal events or events occurring in a critical area such as an intracranial, intra-spinal, intraocular, retroperitoneal, intra-articular or pericardial, or intramuscular event. Bleeding was also defined as major when it occurred in non-critical areas (epistaxis, others) and was associated with a reduction of ≥2 g/dL in the haemoglobin concentration and/or necessitated transfusion of ≥2 blood units. MPN evolution into MF and/or AML was diagnosed according to the current guidelines^10–12^.

### Statistical methods

For continuous variables, the median and the 5th–95th percentiles or range are provided and non-parametric tests for the difference in medians between groups were performed. Categorical variables were summarized by counts and percentages, and groups were compared with a Chi-squared test or Fisher’s exact test where appropriate. The annual incidence rate of any event that occurred during the follow-up was calculated by dividing the number of events by the total number of patient-years.

A composite CV outcome was defined including TIA, non-fatal IS, non-fatal AMI, and CV death, whichever occurred first. The cumulative probability of experiencing a specific outcome during follow-up was estimated by the competing risks method^19^. With this approach, a subject was assumed to experience the outcome only once, and the overall incidence at a given time was split into the sum of the cause-specific cumulative incidences.

Finally, the Cox proportional hazard regression was used to estimate the hazard ratio (HR) and 95% confidence interval (CI) for the association between any potential risk factor and time of each outcome onset. Starting with all candidate variables (i.e., those listed in Table 1 as well as antithrombotic or cytoreductive treatment), backward selection was used to test whether the deletion or retention of each variable improved the model, repeating this process until no further improvement was possible. For all hypotheses tested, two-tailed *p*-values less than 0.05 were considered significant.

**Table 1:** Patient demographic and clinical characteristics

	*N*	Index stroke (*N* = 327)	Index TIA (*N* = 270)	*p*-value
Male/female—*n* (%)	597	141/186 (43/57)	105/165 (39/61)	0.296
Age at index event—median (5th–95th percentile)	597	69 (37–84)	68.5 (40–83)	0.970
MPN characteristics				
MPN diagnosis—*n* (%)	597			0.201
Polycythemia vera		107 (33)	77 (29)	
Essential thrombocythemia		174 (53)	163 (60)	
Myelofibrosis		46 (14)	30 (11)	
Mutational status—*n* (%)	553			0.490
JAK2 mutation/exon 12		270 (89)	211 (85)	
CALR mutation		18 (6)	21 (8)	
MPL mutation		4 (1)	6 (2)	
Triple negatives		12 (4)	11 (4)	
Data at index event				
Imaging—*n* (%)	578			
Computed tomography scan		203 (64)	177 (68)	0.236
Magnetic resonance imaging scan		116 (36)	82 (32)	
Blood cells values—median (5th–95th percentile)				
Hemoglobin (g/dL)	433	14.0 (10.4–17.9)	14.2 (10–17.6)	0.401
Hematocrit (%)	418	43.0 (32.0–55.6)	44.0 (34.0–54.0)	0.238
White blood cells count (×10^9^/L)	440	9.1 (4.5–20.0)	8.9 (5.0–17.1)	0.387
Platelets count (×10^9^/L)	459	523 (204–1067)	551 (255–1103)	0.206
Index event heralding MPN diagnosis^a^—*n* (%)	597	149 (46)	101 (37)	0.044
Index event after MPN diagnosis—*n* (%)		178 (54)	169 (63)	
Years from MPN diagnosis to index event—median (5th–95th percentile)	347	4.37 (0.26–22.2)	4.48 (0.35–16.3)	0.958
Cardiovascular risk factors				
History of remote^b^ arterial and/or venous thrombosis—*n* (%)	597	76 (23)	44 (16)	0.035
History of remote^b^ cerebrovascular thrombosis—*n* (%)	597	25 (8)	16 (6)	0.408
Active smoking	597	82 (25)	46 (17)	0.017
Hypertension	597	187 (57)	140 (52)	0.192
Dyslipidemia	597	91 (28)	55 (20)	0.035
Diabetes	597	38 (12)	30 (11)	0.845
Atrial fibrillation	597	23 (7)	10 (4)	0.079
Other cardiogenic embolisms^c^	597	42 (13)	25 (9)	0.167
Microvascular disturbances^d^	597	77 (24)	83 (31)	0.048
Presence of thrombophilia—*n* (%)	238	54 (43)	40 (36)	0.307
Inherited thrombophilia^e^		16 (13)	13 (12)	0.835
Hyperhomocysteinemia		30 (24)	18 (16)	0.155
Antiphospholipid antibodies^f^		13 (10)	12 (11)	0.885

^a^Index events (ischemic stroke and TIA) occurred within a maximum of two years before the diagnosis of MPN or as heralding manifestation of MPN

^b^Anamnestic thrombosis occurred two years or more before the diagnosis of MPN

^c^Other embolisms or cardiogenic embolisms include patent foramen ovale, valvular heart disease (mitral stenosis, artificial valves), significant carotid artery stenosis, congestive heart failure, and coronary artery disease

^d^Microvascular disturbances include erythromelalgia, scintillating scotoma, pulsatile headache, dizziness, and tinnitus

^e^Inherited thrombophilia includes deficiency of antitrombin, protein C, protein S, factor V Leiden, prothrombin G20210A, increased levels of factor VIII

^f^Antiphospholipid antibodies include lupus anticoagulant, anticardiolipin antibodies, and anti-beta2-glycoprotein I antibodies

## Results

### Clinical and laboratory features of the cohort

The initial cohort suitable for the study consisted of 5102 MPN patients diagnosed from January 2005 through December 2015. Out of 615 patients recorded by centres, 18 were excluded: two with hemorrhagic stroke, and 16 with TIA or IS not considered as MPN-related because occurred before 2 years preceding the diagnosis of MPN, leaving a total of 597 MPN patients with documented TIA (*n* = 270) or IS (*n* = 327).

The majority were over 60 years of age at the time of the index event; the frequency of the *JAK2 V617F* mutation was 100% in PV patients and 82% and 76% in ET and PMF patients, respectively.

IS was documented more frequently than TIA at diagnosis, while TIA occurred in 63% of the cases during the course of MPN disease (Table 1). The distribution of PV, ET and PMF and baseline blood counts were similar in IS and TIA groups; patients with IS had more often a history of remote vascular events (more than 2 years before MPN diagnosis), active smoking, dyslipidaemia and atrial fibrillation, while TIA cases had a significantly higher history of microvascular disturbances (Table 1).

Even though thrombophilia had a similar occurrence in the two groups, a trend for a greater frequency of inherited abnormalities was seen in patients less than 40 years (25%) than in the other age categories (13% and 9% for age 40–60 years and over 60 years, respectively; *p* = 0.094).

Almost all patients received cytoreductive therapy (hydroxyurea in the large majority) and long-term treatment with low-dose aspirin particularly after TIA (*p* < 0.0001), while oral anticoagulants were more frequently prescribed after stroke (*p* < 0.0001) (Table 2).

**Table 2:** Medication use after the index stroke or TIA

	Index stroke (*N* = 313^a^)	Index TIA (*N* = 264^a^)	*p*-value
Antithrombotic treatment			
None	14 (4)	10 (4)	0.681
Antiplatelet agent	247 (79)	239 (91)	<0.0001
Single agent (ASA or other)	235 (75)	230 (87)	<0.0001
Double agent (ASA + other)	12 (4)	9 (3)	0.786
Anticoagulant agent	51 (16)	14 (5)	<0.0001
VKA	48 (15)	13 (5)	<0.0001
DOAC	3 (1)	1 (<1)	0.629
Heparin	1 (<1)	1 (<1)	0.904
Cytoreductive treatment			
None	32 (10)	25 (9)	0.762
Phlebotomy	2 (<1)	8 (3)	0.049
Hydroxyurea	248 (79)	204 (77)	0.569
Alone	224 (72)	193 (73)	0.680
In combination	24 (8)	11 (4)	0.079
Anagrelide	3 (1)	15 (6)	0.001
Interferon	11 (4)	6 (2)	0.380
Ruxolitinb	5 (2)	3 (1)	0.733
Other^b^	12 (4)	3 (1)	0.063

Overall, 503 patients (87.2%) received either cytoreductive and antithrombotic treatments; 50 patients (8.7%) received only antithrombotic treatment (and no cytoreductive treatment), 17 patients (2.9%) received only cytoreductive treatment (and no antithrombotic treatment), and 7 patients (1.2%) received neither cytoreductive nor antithrombotic treatments

*ASA* acetylsalicylic acid, *VKA* vitamin K-antagonist, *DOAC* direct oral anticoagulant

^a^Twenty out of 597 patients (3%) have missing information on treatments after the index event

^b^Other cytoreductive treatment includes pipobroman, busulphan, P32

### Single and composite CV outcomes

During a total follow-up time of 2347 years after the index event (median 4.2, range 0–11.9), 113 patients (18.9%) experienced an incident thrombosis, 76% in arterial and 24% in venous sites (Table 3). The incidence rates of overall de-novo thrombosis and recurrent ICVE were 4.81 (95% CI 3.91–5.78) and 2.59 (95% CI 1.98–3.33).

**Table 3:** Overall incidence of major events after the index stroke or TIA

	Index stroke (N = 327)			Index TIA (N = 270)			*p*-value^a^
	Events, *n* (%)	Patient-years	Incidence rate % pt-yrs (95% CI)	Events, *n* (%)	Patient-years	Incidence rate % pt-yrs (95% CI)	
Thrombotic events, *n* = 113	54 (17)	1179	4.41 (3.36–5.79)	59 (22)	1168	4.97 (3.84–6.42)	0.5350
Arterial thrombosis, *n* = 86	38 (12)	1228	3.10 (2.25–4.25)	48 (18)	1199	3.92 (2.94–5.22)	0.2814
Unstable angina pectoris	1 (0.3)			1 (0.4)			
Acute myocardial infarction	4 (1.2)	1308	0.31 (0.11–0.82)	10 (3.7)	1350	0.67 (0.35–1.28)	0.1982
TIA^+^	13 (4.0)	1290	1.01 (0.59–1.74)	29 (10.7)	1243	2.33 (1.62–3.36)	0.0098
Stroke^+^	15 (4.6)	1291	1.6 (0.70–1.93)	4 (1.5)	1378	0.29 (0.11–0.77)	0.0079
Peripheral artery thrombosis	4 (1.2)			4 (1.5)			
Splenic infarction	1 (0.3)			0 (0)			
^ +^TIA + stroke, *n* = 61	28 (9)	1255	2.23 (1.54–3.23)	33 (12)	1240	2.66 (1.89–3.74)	0.4959
Venous thrombosis, *n* = 27	16 (5)	1278	1.10 (0.65–1.85)	11 (4)	1349	0.82 (0.45–1.47)	0.4709
DVT of the legs	5 (1.5)			7 (2.6)			
DVT of the legs + PE	3 (0.9)			2 (0.7)			
Pulmonary embolism	2 (0.6)			0 (0)			
Superficial vein thrombosis	2 (0.6)			1 (0.4)			
Hepatic vein thrombosis	1 (0.3)			0 (0)			
Splanchnic vein thrombosis	2 (0.6)			0 (0)			
Cerebral vein thrombosis	1 (0.3)			0 (0)			
Retinal vein thrombosis	0 (0)			1 (0.4)			
Major bleeding, *n* = 25	15 (5)	1299	1.08 (0.64–1.82)	10 (4)	1355	0.74 (0.40–1.37)	0.3681
Intracranial bleeding	6 (1.8)			2 (0.7)			
Gastrointestinal bleeding	3 (0.9)			5 (1.9)			
Other sites	6 (1.8)			3 (1.1)			
Evolution to MF, *n* = 19	9 (3)	1308	0.69 (0.36–1.32)	10 (4)	1356	0.66 (0.35–1.28)	0.9407
Evolution to AML, *n* = 17	6 (2)	1326	0.45 (0.20–1.01)	11 (4)	1380	0.80 (0.44–1.44)	0.2713
Second neoplasia, *n* = 51	26 (8)	1273	2.04 (1.39–3.00)	25 (9)	1341	1.79 (1.20–2.67)	0.6435
Breast	5 (1.5)			5 (1.9)			
Gastrointestinal/liver	3 (0.9)			5 (1.9)			
Kidney	0 (0)			2 (0.7)			
Lung	1 (0.3)			1 (0.4)			
Lymphoma/CLL	3 (0.9)			2 (0.7)			
Prostate/bladder	5 (1.5)			2 (0.4)			
Skin	4 (1.2)			4 (1.5)			
Other	5 (1.5)			4 (1.5)			
Death, *n* = 102	61 (19)	1327	4.37 (3.38–5.66)	41 (15)	1381	2.97 (2.19–4.03)	0.0572
Cardiovascular	26 (8.0)	1327	1.73 (1.15–2.61)	8 (3.0)	1381	0.58 (0.29–1.16)	0.0050
Infection	4 (1.2)			4 (1.5)			
MF	1 (0.3)			1 (0.4)			
AML	6 (1.8)			8 (3.0)			
Second neoplasia	4 (1.2)			7 (2.6)			
Hemorrhage	0 (0)			1 (0.4)			
Unknown	20 (18.7)			12 (4.4)			

^a^Tests for differences in incidence rates

The cumulative incidence of single and composite outcome is reported in Table 4. At one year, the cumulative incidence of the composite CV outcome was lower, though not significant, after TIA (4.21%) than after IS (7.14%) (*p* = 0.13), but the frequency resulted similar after 5 years (18.9 and 19.2%). The lower frequency of a composite outcome in the first year after TIA was due to the absence of incident cases of strokes and AMI, while recurrent TIA and AMI progressively increased at 5 years to 12% and 3.74% of patients, respectively. In contrast, after the index stroke, cerebral events including TIA and new IS occurred earlier and progressively increased. However, after 5 years, the two groups showed a similar cumulative incidence of ischemic cerebrovascular recurrences (13.2% and 12.3% after TIA and stroke, respectively), although the occurrence of the single outcome was different.

**Table 4:** Cumulative incidence of major events after the index stroke or TIA

	TIA % (95% CI)	Non-fatal stroke % (95% CI)	Non-fatal AMI % (95% CI)	CV mortality % (95% CI)	Composite CV outcome^a^ % (95% CI)	Non-fatal venous thrombosis % (95% CI)	Overall mortality % (95% CI)
Index TIA							
30-day	0.37 (0.05–2.61)	–	–	0.37 (0.05–2.60)	0.74 (0.19–2.93)	–	0.37 (0.05–2.60)
90-day	1.49 (0.56–3.92)	–	–	0.37 (0.05–2.60)	1.86 (0.78–4.40)	–	0.37 (0.05–2.60)
6-month	1.87 (0.78–4.44)	–	–	0.75 (0.19–2.96)	2.61 (1.25–5.40)	–	1.13 (0.37–3.46)
12-month	3.48 (1.82–6.58)	–	–	0.75 (0.19–2.96)	4.21 (2.35–7.47)	0.79(0.20–3.12)	1.92 (0.80–4.56)
24-month	6.57 (4.07–10.5)	–	2.65 (1.20–5.80)	1.65 (0.62–4.35)	10.80 (7.47–15.5)	1.24 (0.40–3.80)	5.38 (3.15–9.09)
60-month	12.0 (8.26–17.2)	1.24 (0.31–4.95)	3.74 (1.88–7.40)	2.14 (0.89–5.08)	18.9 (14.2–24.9)	3.68 (1.74–7.73)	10.0 (6.69–14.9)
Index stroke							
30-day	–	0.31 (0.04–2.20)	0.32 (0.04–2.23)	1.55 (0.65–3.68)	2.17 (1.04–4.50)	0.63 (0.16–2.51)	1.55 (0.65–3.68)
90-day	–	0.96 (0.31–2.95)	0.32 (0.04–2.23)	2.18 (1.04–4.51)	3.44 (1.92–6.12)	1.61 (0.67–3.83)	2.18 (1.04–4.51)
6-month	0.67 (0.17–2.65)	0.96 (0.31–2.95)	0.32 (0.04–2.23)	2.50 (1.26–4.94)	4.41 (2.64–7.34)	1.94 (0.88–4.27)	2.50 (1.26–4.94)
12-month	2.04 (0.92–4.49)	2.03 (0.92–4.47)	0.67 (0.17–2.64)	2.50 (1.26–4.94)	7.14 (4.75–10.6)	1.94 (0.88–4.27)	2.84 (1.49–5.40)
24-month	2.86 (1.44–5.66)	3.61 (1.96–6.63)	0.67 (0.17–2.64)	3.64 (2.02–6.49)	10.2 (7.23–14.3)	2.76 (1.38–5.46)	6.67 (4.29–10.3)
60-month	5.81 (3.32–10.1)	6.52 (3.90–10.8)	1.80 (0.65–4.91)	7.18 (4.52–11.3)	19.2 (14.6–25.0)	4.60 (2.46–8.49)	20.4 (15.4–26.6)

*TIA* transient ischemic attack, *AMI* acute myocardial infarction, *CV* cardiovascular

^a^Composite CV outcome includes non fatal TIA, Stroke, AMI, and CV deaths, whichever occurs first

**Fig. 1:**
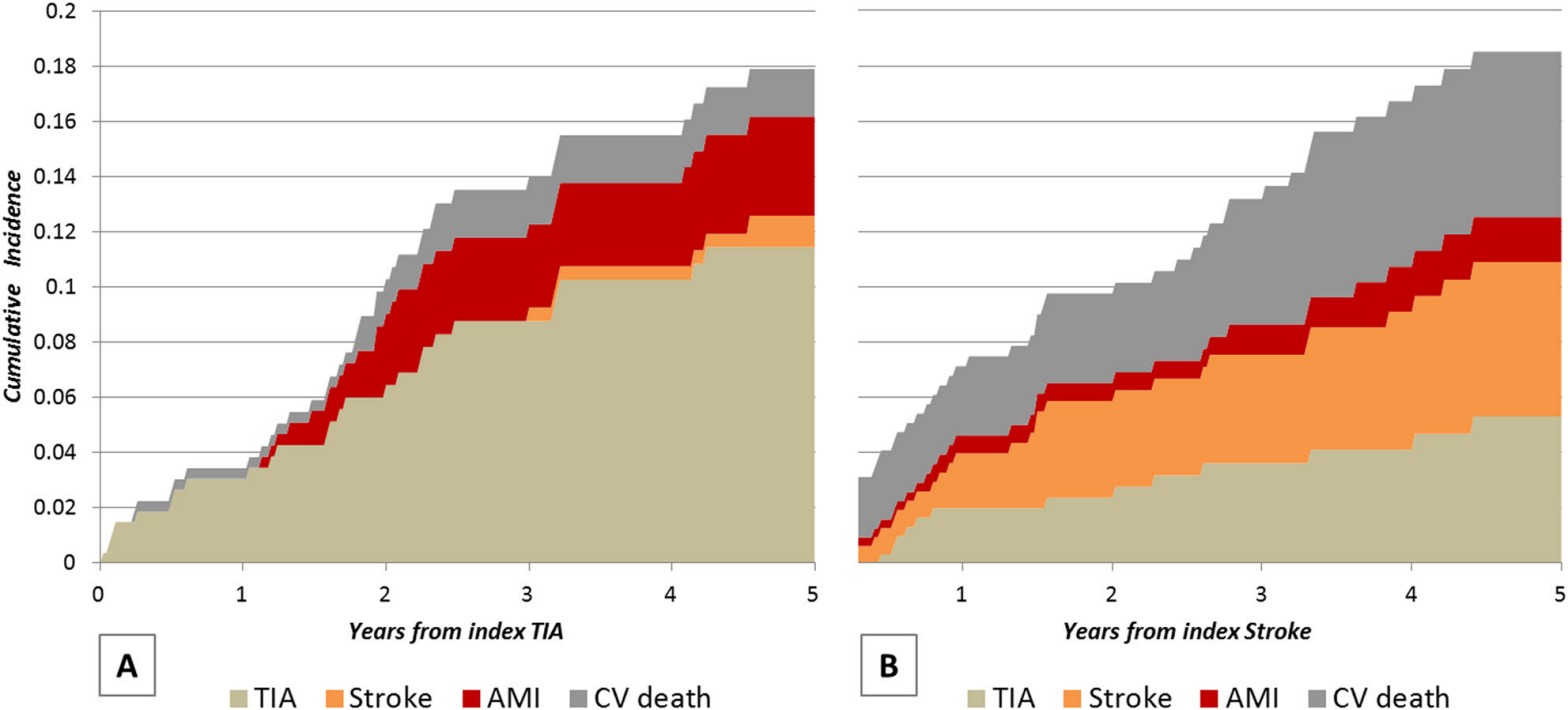
Cumulative incidence of TIA, non-fatal stroke, non-fatal AMI, and cardiovascular (CV) death by type of index event (panel a: index TIA; panel b: index stroke). The cumulative probability of experiencing a specific outcome was estimated by the competing risks method

In TIA patients, recurrent TIA contributed to 63% of the composite CV outcome; in contrast, the single outcomes after IS (CV mortality, IS, and TIA) contributed equally to the estimate of the composite outcome; in particular, after 5 years, CV deaths were more frequent than after TIA (7.18 and 2.14% respectively, *p* = 0.011). Figure 1 offers a graphic representation of the cumulative incidence of the specific outcomes contributing to the composite CV outcome after the two index events.

Venous thrombosis showed a higher cumulative incidence in the first year after stroke than after TIA (*p* = 0.03 and *p* = 0.02 at 3 and 6 months, respectively), but these rates were similar after 5 years (3.68% and 4.60%, respectively).

The overall mortality after 1 year was comparable in the two groups (2.84% vs. 1.92%) and was due to CV complications in 88% after stroke and 39% after TIA. After 5 years, deaths for CV causes decreased to 35% after stroke and to 21% after TIA. Other causes of death were due to MF, acute leukaemia, solid tumour, infections, or haemorrhages (Table 3).

**Fig. 2:**
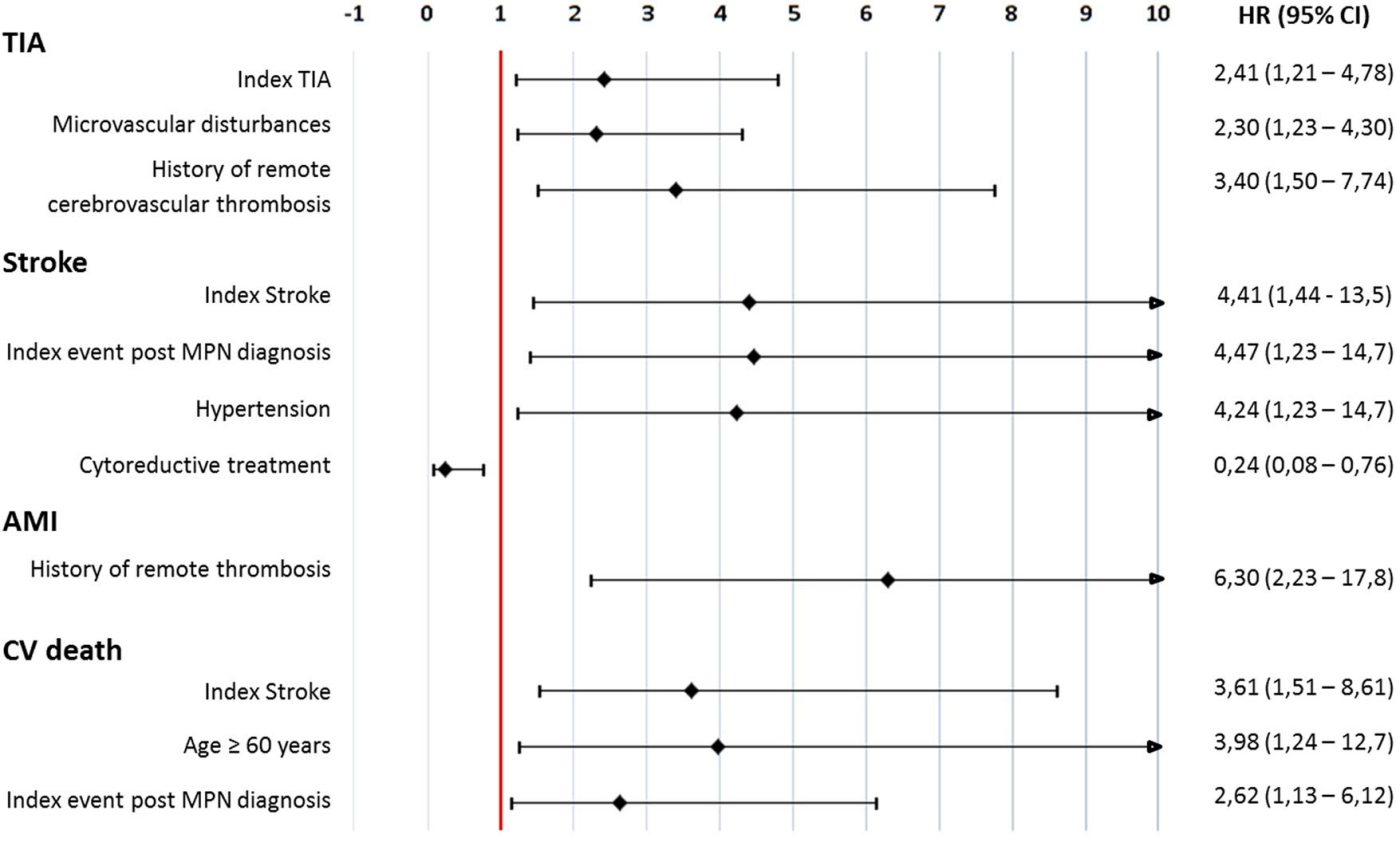
Hazard ratio (and 95% confidence interval) for the association between significant risk factors and time of CV outcomes onset. Estimates from multivariate Cox proportional hazard models with competitive risks method. Significant (*p* < 0.05) hazard ratio (HR) estimates kept by backward selection beginning from the full model on all explanatory variables are plotted

The significant variables associated with arterial events, identified by backward selection for each specific outcome starting from the full model including all explanatory variables, are presented in Fig. 2. Independent prognostic factors of incident recurrent TIA were a previous index TIA, history of remote TIA and microvascular disturbances. Likewise, new incident ISs were predicted by an index stroke, particularly occurring after MPN diagnosis. In the IS group, arterial hypertension emerged as an independent risk factor. Of note, cytoreductive treatment was a strong protective factor capable of reducing the risk of new IS in 76% of patients. The only prognostic risk factor for AMI was a prior history of remote arterial and venous thrombosis, while CV death was predicted by a previous index IS, age over 60 years, and by the index event occurring after MPN diagnosis.

### Bleeding

Major bleeding occurred in 25 patients (4%), corresponding to an incidence rate of 0.9 per 100 patient-years (Table 5), with no difference between patients receiving antiplatelet drugs or oral anticoagulants. Of note, the eight cases experiencing cerebral bleeding were receiving single agent aspirin while no events in critical areas were seen in patients receiving oral anticoagulants. Among the risk factors, univariate analysis showed that age over 75 year was significantly associated with a risk of major haemorrhages (HR = 2.39, 95% CI 1.07–5.34, *p* = 0.035); however, in the multivariate model, corrected by the already mentioned risk factors, this effect was reduced to a trend (*p* = 0.074), while significant factors were the history of remote thrombosis (HR = 2.51, 95% CI 1.05–5.96, *p* = 0.038) and the thrombotic complications arising after MPN diagnosis (HR = 2.99, 95% CI 1.08–8.29, *p* = 0.035).

**Table 5:** Incidence of major bleeding according to antithrombotic therapy

	N (%)	Total, *N* = 597 % pts/year (95% CI)	Antiplatelet agents, *n* = 486 % pts/year (95% CI)	Anticoagulant agents, *n* = 65 % pts/year (95% CI)	*p*-value^a^
Major bleeding	25 (4)	0.90 (0.61–1.35)	0.94 (0.61–1.44)	0.79 (0.20–3.16)	0.980
CNS bleeding	8 (1.3)	0.30 (0.15–0.59)	0.35 (0.18–0.70)	0 (0)	0.426
GI bleeding	8 (1.3)	0.26 (0.12–0.55)	0.26 (0.12–0.59)	0.40 (0.06–2.81)	0.674
Muscle hematoma	5 (0.8)	0.19 (0.08–0.45)	0.18 (0.07–0.47)	0.39 (0.05–2.77)	0.497
Epistaxis	4 (0.6)	0.15 (0.06–0.40)	0.13 (0.04–0.41)	0 (0)	0.726

*CNS* cerebral nervous system, *GI* gastrointestinal

^a^Tests for differences in incidence rate between antiplatelet agents and anticoagulant agents groups

## Discussion

The large cohort of MPN patients included in the PRISM study represents, to the best of our knowledge, the most updated and comprehensive clinical epidemiological picture of how patients with cerebrovascular ischemia are managed in specialized haematological centres. In this large cohort, we obtained for the first time an accurate and comprehensive picture of the history of incident recurrences, CV events, and death over long-term follow-up after the occurrence of an index TIA and IS.

At index TIA and IS presentation, patients had atrial fibrillation in a minority of cases (4 and 7%) compared with the general population (11–18% in TIA cases and 20–30% in IS cases, respectively)^20–23^; dyslipidemia and diabetes were also less frequent, accounting for approximately 50% of those found in non-MPN patients^20–23^. Therefore, the association of these comorbidities with TIA and IS events appears less evident in MPN than in non-MPN patients, suggesting that the condition of thrombophilia due to the underlying disease plays a major role via constitutive activation of the haemostatic system^4^. We underline the high frequency of *JAK2V617F* mutated patients in our cohort, confirming the significant influence of the *JAK2V617F* mutation in the pathogenesis of thrombosis in these clonal diseases^4^. Of interest, a great proportion of the patients presenting with an index TIA and IS had a prior history of microvascular disturbances underscoring their possible significance as predictors of major thrombosis in MPN^24^.

To prevent recurrences and CV mortality, the patients in this study had received aspirin, vitamin K antagonists (VKA) or direct oral anticoagulants as recommended for the general population^25^, in addition to cytoreductive drugs according to the MPN treatment guidelines^10–12^.

At one year after the TIA index event, 51 outcomes (8 deaths from CV causes, 4 IS, 10 AMI, and 29 TIA), corresponding to a composite cumulative incidence of 4.21%. This compares favourably with a contemporary cohort of patients with TIA or minor stroke recruited from the general population, in which the composite outcome at one year was 6.2%^22^. However, in contrast to the general population, the single outcomes after TIA presented with a different incidence. It is remarkable that among patients with TIA, no IS occurred in the first 2 years and that strokes occurred after 5 years in only 1.24% of cases. These estimates are lower than in contemporary non-MPN patients with TIA, in whom strokes occurred in 4.4–5.1% of patients after 1 year^14,15,22^, and 12–13.2% after 5 years^14,15^. In contrast, while strokes were rare events, the cumulative incidence of new TIA episodes progressively increased year by year and reached a 12% incidence after 5 years, with a 10% overall mortality rate. On the other hand, after the index IS, new strokes occurred with a cumulative incidence lower than in the general population: after 1 and 5 years, it was 2.03 and 6.52% *vs* 11.1–12% and 26.4% of non-MPN patients, respectively^16,23^. As expected, in comparison with TIA, overall mortality after stroke was doubled (20.4%), due to CV causes in 35% of cases. As regards risk factors for recurrent events, TIA or IS patients tended to replicate the first manifestation (HR for recurrent TIA and recurrent IS 2.4 and 4.4, respectively); moreover recurrent TIA was significantly anticipated by microvascular symptoms (HR 2.3), whereas recurrent stroke was associated with hypertension (HR 4.2). The use of cytoreductive drugs was a strong protective factor and decreased the rate of recurrent IS by 76%. Notably, the occurrence of the index events in the course of follow-up after diagnosis of MPN, in spite of antithrombotic prophylaxis and cytoreductive drugs, was identified as a strong prognostic factor for IS and CV death.

The different clinical course of recurrences after index TIA or IS could reflect a non-univocal pathogenesis of the two clinical phenotypes. We speculate that in TIA, there is a prominent role of haemostatic alterations characterized by an ongoing process of platelet activation, platelet aggregation and abnormal platelet-endothelial interactions, producing endothelial cell activation and transient damage in microvascular districts^26–29^. This haemostatic perturbance, when combined with hypertension, was found to be independently associated with recurrences in patients with IS, reflecting a condition leading to a higher frequency of more severe cerebral ischemic events.

An important outcome that should be evaluated after TIA and stroke is myocardial infarction. The frequency at 5 years of AMI was 3.74% and 1.80% in TIA and stroke, respectively, which are estimates comparable to the general population^30^. Finally, the cumulative incidence of venous thromboembolism (VTE) was higher after stroke in the first year, while at 5 years it was comparable after TIA and stroke (3.68% and 4.60%, respectively). This latter estimate is strikingly lower than that reported in a population-based study in which the cumulative incidence of VTE after stroke was as high as 15% at 3 months after the event, with a subsequent slow increase up to approximately 40% at 5 years after a stroke^31^. All in all, the cumulative incidence of the composite outcome, including ICVE, AMI, and CV deaths, was the same (19%) at 5 years after both an index TIA and IS, even though the single outcomes were differently represented.

The limitations of our study are those of a retrospective observational study. Case ascertainment, however, was based on objective imaging in almost the totality of cases (97%) and confirmation of the diagnosis by experienced stroke clinicians who took care of the patients in the acute phase. The collection of the data was made by expert haematologists and every effort was made to minimize missing data and incomplete follow-up; as a result, no data concerning traditional CV risk factors were missing, and no patient was lost to follow-up. The patients were selected from an MPN database of over 5000 patients diagnosed by expert centres in the last decade, so that our results can be generalized to the overall contemporary population of the MPN patients.

At least three reasons may account for the inferior rate of CV complications after TIA or strokes in MPN compared to the general population: first, atrial fibrillation is recognized as a risk factor for recurrent ICVE^32^, and this condition was low in our patient population; second, we speculate about a better control and adherence to the prescribed drugs (antithrombotic, anti-hypertensive, statins, and antidiabetics) due to the more frequent follow-up visits needed to monitor chemotherapy; and third, we underline the antithrombotic efficacy of hydroxyurea on MPN-associated risk factors^33,34^. The antithrombotic effect of hydroxyurea may recognize additional mechanisms of action besides myelosuppression, including qualitative changes in leukocyte function, decreased expression of endothelial adhesion molecules, and enhanced nitric oxide generation^35,36^.

The favourable risk-benefit profile of therapy in the prevention of CV events was obtained without enhancing the risk of major bleeding, which had an incidence similar to the general population receiving long-term antiplatelet secondary prophylaxis^37^.

Even though the recurrent events after TIA and stroke showed an inferior incidence compared to the non-MPN population, a residual risk of new TIA and strokes remained, and future studies should be designed to test new antithrombotic therapy and perhaps include new cytoreductive drugs.

However, the long-term observation of this cohort suggests some considerations that may have practical clinical relevance: (a) MPN patients with a previous ICVE are prone to recurrence including also other major CV events; (b) the observed advantage for recurrence when compared to the general population, is likely due to a strict adherence to the recommended treatment options for long-term treatment of MPN patients with thrombosis^10–12^, based on antithrombotic treatment, cytoreduction, and phlebotomies; (c) cytoreduction with hydroxyurea resulted particularly effective in patients with IS, who are at higher risk of CV death; (d) although the incidence of major bleeding was aligned with that expected in the general population under anticoagulants, a prudent use of aspirin may be suggested in patients with a previous long history of treatments for thrombosis and with age over 75 years, given the enhanced hemorrhagic risk^37^.

In conclusion, current antithrombotic therapy and cytoreductive drugs after an index TIA and stroke in MPN lead to a reduction of cardio-vascular recurrences and mortality in a remarkable proportion of patients, resulting even superior to that in the general population. Overall, the benefit-risk profile of treatment with antithrombotic treatment and cytoreduction in these neoplastic disorders appears favourable, but some categories of patients with TIA and stroke occurring after MPN diagnosis might require special strategies, to be assessed in future studies.
